# Primary immunodeficiency

**DOI:** 10.1186/s13223-018-0290-5

**Published:** 2018-09-12

**Authors:** Christine McCusker, Julia Upton, Richard Warrington

**Affiliations:** 1 McGill University, Montreal, QC Canada; 2 Division of Immunology and Allergy, Hospital for Sick Children, Toronto, ON Canada; 3 Department of Paediatrics, University of Toronto, Toronto, ON Canada; 4 University of Manitoba, Winnipeg, MB Canada

## Abstract

Primary immunodeficiency disorder (PID) refers to a large heterogeneous group of disorders that result from defects in immune system development and/or function. PIDs are broadly classified as disorders of adaptive immunity (i.e., T cell, B-cell or combined immunodeficiencies) or of innate immunity (e.g., phagocyte and complement disorders). Although the clinical manifestations of PIDs are highly variable, many disorders involve an increased susceptibility to infection. Early consultation with a clinical immunologist is essential, as timely diagnosis and treatment are imperative for preventing significant disease-associated morbidity. PIDs should be suspected in patients with: recurrent sinus or ear infections or pneumonias within a 1 year period; failure to thrive; poor response to prolonged use of antibiotics; persistent thrush or skin abscesses; or a family history of PID. Patients with multiple autoimmune diseases should also be evaluated. Diagnostic testing often involves lymphocyte proliferation assays, flow cytometry, measurement of serum immunoglobulin (Ig) levels, assessment of serum specific antibody titers in response to vaccine antigens, neutrophil function assays, stimulation assays for cytokine responses, and complement studies. The treatment of PIDs is complex and generally requires both supportive and definitive strategies. Ig replacement therapy is the mainstay of therapy for B-cell disorders, and is also an important supportive treatment for many patients with combined immunodeficiency disorders. The disorders affecting the activity of the T-cell arm of the adaptive system, such as severe combined immunodeficiency, require immune reconstitution as soon as possible. The treatment of innate immunodeficiency disorders varies depending on the type of defect, but may involve antifungal and antibiotic prophylaxis, cytokine replacement, vaccinations and bone marrow transplantation. This article provides an overview of the major categories of PIDs and strategies for the appropriate diagnosis and management of these rare disorders.

## Background

Primary immunodeficiency disorder (PID) refers to a heterogeneous group of disorders characterized by poor or absent function in one or more components of the immune system which predisposes affected individuals to increased frequency and severity of infection, autoimmunity, and aberrant inflammation and malignancy. More than 250 different disorders have been genetically identified to date, with new disorders continually being recognized [1, 2]. Most PIDs result from inherited defects in immune system development and/or function; however, acquired forms have also been described [3, 4], such as neutralizing anti-interferon-γ autoantibody-associated immunodeficiency (which has been noted in over 95% of patients with disseminated infections by nontuberculous mycobacteria) [4]. It is important to note that PIDs are distinct from secondary immunodeficiencies that may result from other causes, such as viral or bacterial infections, malnutrition, immunoglobulin (Ig) loss, malignancy or treatment with drugs that induce immunosuppression [5–7].

With the exception of immunoglobulin A (IgA) deficiency, the estimated overall prevalence of these disorders in the United States is approximately 1 in 1200 live births. IgA deficiency is the most common PID, occurring in approximately 1 in 300 to 1 in 500 persons [8].

The clinical presentation of PIDs is highly variable; however, most disorders involve increased susceptibility to infection. In fact, many PIDs present as “routine” infections (often of the sinuses, ears and lungs) and, therefore, may go undetected in the primary-care setting. PIDs may present at any age, and the accurate and timely diagnosis of these disorders requires a high index of suspicion and specialized testing. Therefore, consultation with a clinical immunologist who is experienced in the evaluation and management of immunodeficiencies is essential, since early diagnosis and treatment are critical for preventing significant disease-associated morbidity and improving patient outcomes [9, 10]. This article provides an overview of the major categories of PIDs as well as strategies for the timely identification, diagnosis and management of these disorders.

## Classification

PIDs are broadly classified according to the component of the immune system that is primarily disrupted: adaptive or innate immunity (see *An Introduction to Immunology and Immunopathology* in this supplement for more information on adaptive and innate immunity). The current view of PIDs includes an increasing number of syndromes that are associated with autoimmunity and immune dysregulation as predominant features, rather than an overt pathological risk of infections [11]. This concept of immune dysregulation as “immunodeficiency” is novel and will become increasingly important in the field in the coming years. Table 1 presents a simplified classification highlighting the major categories of PIDs [1–3, 9].

**Table 1:** Simplified classification of PIDs: examples and typical clinical presentations. [1–3, 9, 19]

Classification and examples	Clinical presentation
Disorders of adaptive immunity	
***T-cell (cellular) immunodeficiency***	
• IFN-γ/IL-12	Atypical mycobacterial and salmonella infections
• AIRE mutations	Mucocutaneous candidiasis (thrush) and autoimmune endocrinopathy
***B-cell (antibody-mediated) immunodeficiency***	
• XLA	
• CVID	
• Selective IgA deficiency	Recurrent sinopulmonary infections with encapsulated bacteria
• Specific antibody deficiency	Autoimmune disease and increased risk of malignancy in CVID
• IgG subclass deficiency	
***CID***	
• Wiskott-Aldrich syndrome	Thrombocytopenia with bleeding and bruising; eczema; recurrent bacterial and viral infections; autoimmune disease
• Ataxia telangiectasia	Chronic sinopulmonary disease; cerebellar ataxia (difficulty with control of movement); small, dilated blood vessels of the eyes and skin; malignancy
• DiGeorge syndrome	Hypoparathyroidism; seizures; cardiac abnormalities; abnormal facies; infection
• Hyper IgE syndrome	Chronic dermatitis; recurrent, severe lung infections; skin infections; bone fragility; failure to shed primary teeth
• SCID	
T^−^, B^+^	
▪ γc deficiency	
▪ JAK3 deficiency	Severe, recurrent opportunistic infections; failure to thrive; diarrhea; rash
T^−^, B^−^	
▪ ADA deficiency	
▪ RAG 1/2 deficiency	

Disorders of innate immunity	
***Phagocyte defects***	
▪ Chronic granulomatous disease	Severe infection; abscesses with granuloma formation
▪ Leukocyte adhesion deficiency	Recurrent, severe bacterial infections; poor wound healing; delayed separation of the umbilical cord
***Complement defects***	
▪ Deficiency in early complement pathway components (C1q, C1r, C2, C4)	SLE–like syndrome, rheumatoid disease, multiple autoimmune diseases, infections
▪ Deficiency in late complement pathway components (C5, C6, C7, C8, C9)	Neisserial infections, SLE-like syndrome
▪ C3 and regulatory components	Recurrent infections with encapsulated bacteria

Disorders of immune dysregulation	
• HLH	Fever, splenomegaly, cytopenia, rash
• ALPS	Splenomegaly, adenopathy
• IPEX	Autoimmune enteritis, early onset diabetes, thyroiditits, hemolytic anemia, thrombocytopenia, eczema
• APECED	Autoimmunity affecting parathyroid, adrenal, other endocrine organs; candidiasis; dental enamel hypoplasia

*AIRE* autoimmune regulator, *CVID* common variable immunodeficiency, *IgG* immunoglobulin G, *IgE* immunoglobulin E, *IgA* immunoglobulin A, *IFN*γ interferon-γ, *IL* interleukin, *CID* combined immunodeficiency, *SCID* severe combined immunodeficiency, *XLA* X-linked agammaglobulinemia, *SLE* systemic lupus erythematosus, *JAK3* Janus kinase 3, *ADA* adenosine deaminase, *RAG* recombination activating gene, *HLH* hemophagocytic lymphohistiocytosis, *ALPS* autoimmune lymphoproliferative syndrome, *IPEX* immunodysregulation polyendocrinopathy enteropathy X-linked, *APECED* autoimmune polyendocrinopathy candidiasis and ectodermal dystrophy

### Disorders of adaptive immunity

T cells and B cells are the primary cells of the adaptive immune system. B cells mediate antibody production and, therefore, play a major role in antibody-mediated (humoral) immunity. T cells, on the other hand, govern cell-mediated immune responses. Defects occurring at any stage of T-cell development, differentiation and maturation lead to T-cell (cellular) immunodeficiency disorders, while defects relating to B-cell development and/or maturation result in B-cell (antibody-deficiency) disorders. Since B-cell-mediated antibody production requires intact T-cell function, most T-cell defects lead to combined (B- and T-cell) immunodeficiency disorders (CIDs) [3, 9].

### Disorders of innate immunity

Innate immune responses represent the first line of defense against potential pathogens. Appropriate recognition of threats and induction of the inflammatory cascade are essential steps in the removal of these organisms from the system. Failure of the innate system to identify pathogens delays the induction of the immune response and may worsen outcomes of infection.

Numerous cells and proteins are involved in the innate immune response including phagocytes (neutrophils and macrophages), dendritic cells, and complement proteins. Phagocytes are primarily responsible for phagocytosis, the process by which cells engulf and eliminate invading pathogens. Complement proteins function to identify and opsonize (coat) foreign antigens, rendering them susceptible to phagocytosis. Defects in the development and function of any of these elements of innate immunity may lead to PIDs.

## Clinical presentation

### T-cell and combined immunodeficiencies

The clinical manifestations of T-cell (cellular) disorders and CIDs will vary depending on the specific underlying defect in the adaptive immune response. Therefore, clinical suspicion is important for timely diagnosis of these disorders. Patients with specific T-cell defects may be lymphopenic (i.e., have abnormally low levels of lymphocytes) and neutropenic (i.e., have abnormally low levels of neutrophils). In the most severe forms of CID (also known as severe combined immunodeficiency [SCID]), there is a virtual lack of functional T cells and immune function. These disorders are rare and are generally categorized into whether there is an absence of T cells, but presence of B cells (T^−^, B^+^), or an absence of both T and B cells (T^−^, B^−^) (see Table 1). Natural killer (NK) cell numbers are also informative for determining the genetic phenotype of SCID [3, 9]. However, normal T-cell numbers do not exclude the possibility of T-cell defects, and in patients with clinical presentations consistent with immunodeficiency, further investigations of T-cell function are warranted.

Patients with SCID usually present within the first year of life with chronic diarrhea and failure to thrive; severe, recurrent infections with opportunistic pathogens (e.g., *Candida albicans* [thrush], *Pneumocystis jiroveci*, or cytomegalovirus); and skin rashes. Some patients may also have associated neurological defects. SCID is a pediatric emergency since infection often leads to death and hematopoietic stem cell transplantation (HSCT) can be curative [3, 9].

Other, less severe CIDs that do not characteristically lead to early mortality include Wiskott-Aldrich syndrome, DiGeorge syndrome, ataxia-telangiectasia, and X-linked lymphoproliferative disease. Patients with these disorders often present later in childhood with recurrent infections and clinical findings that vary depending on the specific syndrome (see Table 1). Autoimmunity and immune dysregulation are also frequent complications associated with these CIDs [3, 9]. In adults, late-onset combined immunodeficiency (LOCID) is an emerging PID which was first described in 2009 [12]. Patients with LOCID have low CD4+ve T-cell numbers and may present with opportunistic infections. Other important manifestations include splenomegaly and granulomas.

Hyper-IgE syndrome is another CID characterized by Staphylococcal infections of the skin, bone, and lungs, bony abnormalities and high IgE levels (see Table 1) [1–3, 9, 13]. It has recently been found to result from a mutation in signal transducer and activator of transcription 3 (STAT3) which affects phagocytic cell recognition of *Staphylococcus* as well as osteoclast function involved in bone remodeling [13].

### B-cell immunodeficiencies

B-cell (antibody-deficiency) disorders are the most common type of immunodeficiencies, accounting for approximately 50% of all PID diagnoses [9]. They comprise a heterogeneous group of disorders characterized by an increased susceptibility to respiratory tract infections with bacteria, particularly *Streptococcus pneumoniae* and *Haemophilus influenzae.* Patients present after 6 months of age with recurrent, and often severe, sino-pulmonary infections such as otitis media, sinusitis, and pneumonia. Diarrhea, fatigue, autoimmune manifestations (particularly autoimmune cytopenias), and hearing loss are also common [14–16]. Patients with humoral deficiency often have reduced or absent serum Ig levels, but may also show normal or increased serum Ig levels with abnormal function. More than 50% of patients with humoral immunodeficiency are diagnosed in adulthood [17], and there is generally a prolonged delay between first presentation and diagnosis since many healthcare providers do not consider PID in their differential diagnosis.

More than 20 antibody-deficiency disorders have been defined to date. The best recognized/most common disorders in this category include: X-linked agammaglobulinemia (XLA; also known as Bruton’s agammaglobulinemia), common variable immunodeficiency (CVID), and selective IgA deficiency. XLA results from a mutation in the Bruton’s tyrosine kinase (Btk) gene, which is responsible for mediating B-cell development and maturation. The disorder is characterized by markedly reduced levels of circulating B-cells and serum IgG, IgA and IgM. Affected males usually present within the first 2 years of life with recurrent sinopulmonary infections and absent lymph nodes and tonsils [9, 15]. CVID is a heterogeneous disorder characterized by markedly reduced serum concentrations of IgG, low levels of IgA and/or IgM, and poor or absent responses to immunization. The disorder affects males and females equally, and usually has a later age of onset than other antibody-deficiency disorders (i.e., > 10 years of age). It is associated with recurrent sinopulmonary infections, autoimmune and granulomatous disease, gastrointestinal complications and an enhanced risk of malignancy (e.g., lymphoma and gastric carcinoma). Some patients may also present with bronchiectasis (irreversible widening of portions of the bronchi resulting from damage to the airway wall), which is a common cause of morbidity and mortality in these patients [9].

Milder antibody-deficiency disorders, such as selective IgA deficiency, are associated with variably low serum levels of an immunoglobulin class or subclass and, in some cases, impairments in specific antibody formation. IgA deficiency, for example, is characterized by low or absent levels of serum IgA in the presence of normal levels of IgG and IgM. Most patients with IgA deficiency are asymptomatic. Among those that are symptomatic, up to one-third experience recurrent infections [9].

### Innate immunodeficiencies

Patients with innate immunodeficiency disorders may present at any age, often with unusual or difficult to eradicate infections. The typical signs and symptoms of phagocyte disorders are severe pyogenic (pus-like) bacterial and fungal infections of the skin, respiratory tract, and internal organs, as well as nail and gingival issues and painful sores around the mouth. Chronic granulomatous disease (CGD) is a phagocyte defect associated with a marked susceptibility to certain bacteria (catalase positive) and fungi.

Of all the PIDs, complement deficiencies account for less than 1% of identified cases. Patients with these disorders tend to present with systemic autoimmune disease that resembles lupus erythematosus or with severe or recurrent infections with encapsulated organisms (see Table 1) [1–3, 9, 13].

### Disorders of immune dysregulation

These PIDs are associated with autoimmune disease due to the dysregulation of the immune system as a whole [18]. In many of these disorders, lymphocytes may be present but dysfunctional, allowing for the development of excessive autoreactivity and resultant autoimmune disease and/or other symptoms of immune dysregulation. Disorders that fall into this category include: hemophagocytic lymphohistiocytosis (HLH), autoimmune lymphoproliferative syndrome (ALPS), immunodysregulation polyendocrinopathy enteropathy X-linked (IPEX), and autoimmune polyendocrinopathy candidiasis and ectodermal dystrophy (APECED) [19].

## Diagnosis

**Fig. 1:**
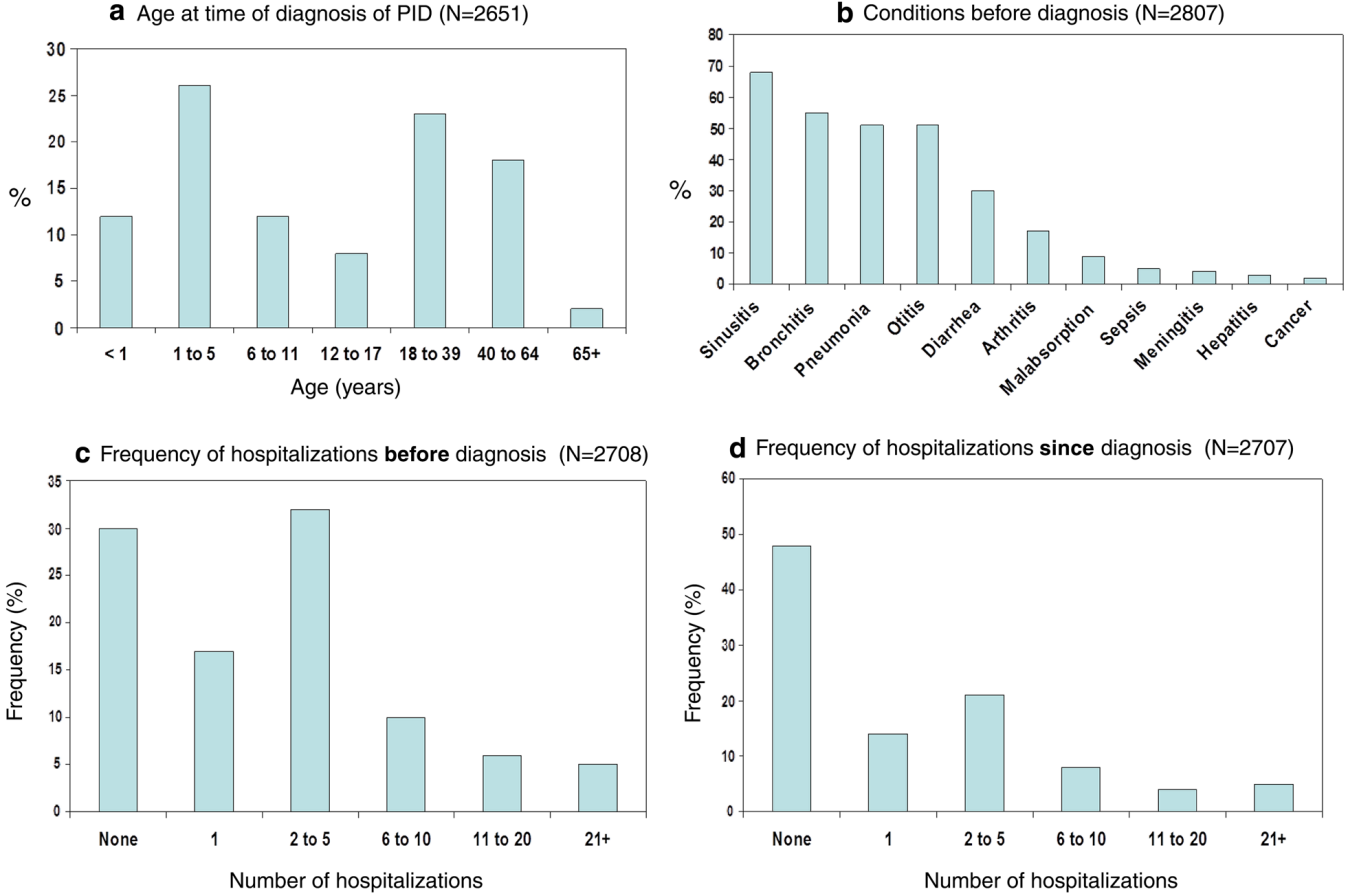
**Results from the Immune Deficiency Foundation (IDF) national survey of PIDs**. Adapted from [20]

As mentioned previously, early diagnosis of PID is critical for preventing significant disease-associated morbidity, and even mortality. However, national surveys of PID conducted by the Immune Deficiency Foundation in the United States found that approximately 60% of patients with these disorders were not diagnosed until adulthood [20, 21] (Fig. 1a), despite the fact that many reported serious or chronic health conditions prior to diagnosis, such as sinusitis, bronchitis, and pneumonia (see Fig. 1b) [20]. The importance of prompt recognition and management of PIDs is further highlighted by the rate of hospitalizations pre- and post diagnosis. Although 70% reported being hospitalized prior to diagnosis (Fig. 1c), nearly half (48%) reported no hospitalization since diagnosis (Fig. 1d) [20].

The difficulty in recognizing SCID in a timely fashion has led to the application of newborn screening to this population. Briefly, when T-cell receptors are generated, a piece of DNA is cut out of the genome which is called a T-cell receptor excision circle (TREC). The TREC count can be used to quantify T-cell production in newborns and will identify many, but not all, cases of SCID. Although not yet available in all jurisdictions, infants identified through newborn screening receive expedited evaluation and, when needed, early HSCT. Outcomes are improved and the risk of complications is reduced when HSCT is initiated early in SCID-affected infants [22].

A diagnosis of PID should be suspected in both children and adults who have recurrent pneumonias and/or ear, sinus and cutaneous infections as listed in Table 2. Although this Table does not provide a comprehensive list of all signs and symptoms of PID, patients meeting any of these criteria should be referred immediately to a clinical immunologist for further evaluation [10, 23]. Other important signs of PID include excessive inflammatory responses and autoimmunity, especially cytopenias. It will also be important for the immunologist to investigate for secondary causes of immunodeficiency, including medications, other infections, immunoglobulin loss and malignancy [5–7].

**Table 2:** The Jeffrey Modell Foundations’ 10 warning signs of primary immune deficiency [23]

**Warning signs in children**
1. ≥ 4 new ear infections within 1 year
2. ≥ 2 serious sinus infections within 1 year
3. ≥ 2 months on antibiotics with little effect
4. ≥ 2 pneumonias within 1 year
5. Failure of an infant to gain weight or grow normally
6. Recurrent, deep skin or organ abscesses
7. Persistent thrush in mouth or fungal infection on skin
8. Need for IV antibiotics to clear infections
9. ≥ 2 deep-seated infections including septicemia
10. A family history of PID
**Warning signs in adults**
1. ≥ 2 new ear infections within 1 year
2. ≥ 2 new sinus infections within 1 year, in the absence of allergy
3. 1 pneumonia per year for > 1 year
4. Chronic diarrhea with weight loss
5. Recurrent viral infections (colds, herpes, warts, condyloma)
6. Recurrent need for IV antibiotics to clear infections
7. Recurrent, deep abscesses of the skin or internal organs
8. Persistent thrush or fungal infection on skin or elsewhere
9. Infection with normally harmless tuberculosis-like bacteria
10. A family history of PID

Adapted from [23]

The immunologist will perform a comprehensive immune evaluation that often begins with a complete blood count (CBC) and blood smear. These tests are used to evaluate for the presence of lymphopenia, abnormal or unusual lymphocytes or phagocytic cells, and any associated gross hematologic abnormalities that may be indicative of PIDs. Significant lymphopenia, for example, may be the first indication of T-cell (cellular) immunodeficiency. Other important diagnostic tools include lymphocyte proliferation assays and flow cytometry which allow for the enumeration of B-cells, T-cells, and NK cells, and the evaluation of lymphocyte markers, T-cell variability, and adhesion receptors that may be associated with specific immune defects. Standard flow cytometry analysis is abnormal in most cases of SCID and in many cases of CID [14, 24, 25].

The initial evaluation of patients with suspected B-cell (antibody-deficiency) disorders involves the measurement of serum IgG, IgA, IgM, and IgE levels (note that the measurement of IgD is not useful for the diagnosis of PIDs). Serum levels that are clearly below age-appropriate reference values may be indicative of B-cell immunodeficiencies. However, some patients with these disorders have normal or only modestly reduced immunoglobulin levels; therefore, the best approach for confirming a diagnosis of an antibody-deficiency disorder is the measurement of serum-specific antibody titers (usually IgG) in response to vaccine antigens. This approach involves immunizing a patient with protein antigens (e.g., tetanus toxoid) and polysaccharide antigens (e.g., pneumococcus) and assessing pre- and post-immunization antibody levels. In many PIDs, antibody responses to these antigens are diminished or even absent [14]. However, interpretation of vaccination response may be challenging. A consensus document from the Basic and Clinical Immunology Interest Section of the American Academy of Allergy, Asthma & Immunology, developed in part using studies in healthy populations, can assist with the application of vaccine responses in the diagnosis of PID [26].

Neutrophil function assays (e.g., dihydrorhodamine 1,2,3 response [DHR]) and stimulation assays for cytokine responses are helpful for confirming a diagnosis of innate disorders. For example, abnormal neutrophil oxidase function is usually indicative of CGD. Complement studies, which examine the level and/or function of specific complement proteins, are essential for the diagnosis of complement deficiency disorders. These studies should be performed by accredited laboratories that have demonstrated competence in these assays and experience in performing investigations into PID [9, 14].

In some cases, more advanced testing for detecting the presence or function of cellular proteins may be used to confirm a diagnosis of PID, and testing for genetic causes is also an important component of diagnosis [27–29]. Initiation of treatment should progress while genetic testing is pursued since many patients with clinical and laboratory evidence of PID do not, as of yet, have an identified single gene defect [24, 25]. Once the diagnosis is established, it is important that therapy be initiated as soon as possible, since delays can lead to permanent organ damage or even death from overwhelming infection [9].

## Treatment

The treatment of PIDs is complex and generally involves both supportive and definitive strategies (see Table 3). As such, therapy should be coordinated by an immunologist with expertise in the management of these disorders [9, 10].

**Table 3:** Strategies for the treatment and management of PIDs

	Supportive	Definitive
**CIDs/SCID**	Ig replacement therapy (IV or SC) Antibiotic prophylaxis Antifungal prophylaxis Aggressive management of established infections Infectious precautions when hospitalized Withhold all live vaccines	BMT HSCT Gene therapy a possibility for some SCIDs

**B-cell disorders**	Ig replacement therapy (IV or SC) Antibiotic prophylaxis Antifungal prophylaxis depending upon etiology Hearing assessment Assessment of pulmonary status and function Close monitoring for co-morbidities	Gene therapy is a potential future treatment in some patients

**Innate disorders**	Antibiotic prophylaxis Antifungal prophylaxis Cytokine replacement (IFNγ) for CGD Vaccinations (e.g., meningococcal) Ig replacement is sometimes indicated	BMT, e.g., for CGD Gene therapy is a potential future treatment

*Ig* immunoglobulin, *IV* intravenous, *SC* subcutaneous, *CID* combined immunodeficiency, *SCID* severe combined immunodeficiency, *IFN*γ interferon-γ, *BMT* bone marrow transplantation, *CGD* chronic granulomatous disease, *HSCT* hematopoietic stem cell transplantation

### SCID/CID

Initial therapy for patients with SCID or other CIDs is supportive and involves aggressive management of the established infection, Ig replacement therapy (discussed in more detail in the next section), and antibiotic and antifungal prophylaxis to reduce the frequency and severity of infections. There is currently no standardized approach to the use of prophylactic antibiotics in patients with established PIDs since randomized, controlled studies in this area are lacking. Commonly used regimens are derived from studies focusing on the prevention of otitis media in children and include: sulfisoxazole, amoxicillin, trimethoprim-sulfamethoxazole (TMP-SMX) and azithromycin [3, 9]. Patients with SCID should also be protected from exposure to infectious agents. In the hospital setting, protective isolation in positive pressure rooms is recommended. Furthermore, live attenuated vaccines (e.g., such as measles/mumps/rubella/varicella, Bacillus Calmette–Guerin, infant rotavirus, and oral polio virus) are contraindicated in patients with SCID as they can lead to severe, disseminated and fatal infections [9]. There is no risk of disseminated infections from killed or inactivated vaccines and, therefore, these may be administered according to routine indications and schedules in patients with PIDs, recognizing that the immune protection gained from these vaccines may be suboptimal.

Since SCID is fatal unless the underlying defect is corrected, definitive therapy with bone marrow transplantation (BMT) or HSCT should be initiated as quickly as possible. When performed from a human leukocyte antigen (HLA)-identical sibling, these procedures lead to excellent long-term survival and long-lasting immune reconstitution. Good results have also been obtained with HLA-mismatched related donors when the procedures are performed within the first 3.5 months of life; however, less satisfactory outcomes have been noted in older patients [3, 9, 30].

Gene therapy, which involves introducing a functional copy of the patient’s defective gene into appropriate cells, has also been shown to lead to immune reconstitution and improved survival in patients with certain SCIDs, such as adenosine deaminase (ADA) deficiency and SCID-X1 (an X-linked inherited SCID characterized by an early block in T-cell differentiation) [31, 32]. Newer techniques for insertion of functional genes are being explored in clinical trials and show significant promise. Enzyme replacement therapy with weekly intramuscular injections of pegylated bovine ADA is also available for the management of patients with ADA deficiency [3].

### B-cell immunodeficiencies

The mainstay of therapy for most B-cell (antibody-deficiency) disorders is intravenous (IV) or subcutaneous Ig replacement therapy; in fact, many patients will require this treatment indefinitely. There are now several sources available for gammaglobulin licensed by Health Canada for use in patients with PID. Table 4 lists some of the subcutaneous and IV immunoglobulin products carried by Canadian Blood Services [33]. However, it is important to note that these products may not be available in all cities/provinces in Canada, and that other products not listed in this table may be available for the treatment of PID. IV and subcutaneous formulations are considered equally effective in reducing the frequency and severity of infections, and there is insufficient evidence to suggest that one product is superior to another, although dosing and frequency of use must be carefully monitored [9, 10, 34]. When deciding on a specific product, patient preference should be taken into consideration. Some patients may prefer a subcutaneous formulation since therapy can be administered at home. Note that intramuscular Ig replacement therapy is not considered to be as effective as IV or subcutaneous therapy and, therefore, is not recommended for the treatment of PID.

**Table 4:** Subcutaneous and IV Ig products carried by Canadian Blood Services [33]

Brand name	GAMUNEX^®^	IGIVnex^®^	Hizentra^™^	Cuvitru^™^
Subcutaneous Ig				
Indications	PID, SID		PID, SID	PID, SID
Concentration	10%		20% solution (200 mg/mL)	20%
IgA content	Average 0.046 g/L		No more than 50 mg/L (avg 8 mg/L)	Average < 80 mcg/mL; no more than 280 mcg/mL
Storage temperature	May be stored for 36 months at + 2 to + 8 °C, and product may be stored at temperatures not to exceed 25 °C (77°F) for up to 6 months anytime during the 36 month shelf life		+ 2 to + 25 °C can be stored either in the refrigerator or at room temperature	+ 2 to + 8 °C
Recommeneded infusion rate	20 mL/hr per infusion site		15 mL/h per site Subsequent infusions, maximum of 34 mL per hour per site as tolerated	≤ 60 mL/hr/site up to 4 sites
Availabe sizes distributed by CBS	2.5 g/25 mL, 5 g/50 mL, 10 g/100 mL, 20 g/200 mL	20g/200 mL	5, 10, 20, 50 mL	1 g/5 mL, 2 g/10 mL, 4 g/20 mL, 8 g/40 mL
Manufacturer website	http://www.grifols.com		http://www.cslbehring.ca	https://www.shirecanada.com

Brand name	Gammagard S/D	GAMUNEX^®^	IGIVnex^®^	Gammagard Liquid	Privigen^®^	Octagam^®^ 10%
IV Ig						
Indications	PID CLL ITP	PID, ITP, SID, CIDP in adults ≥ 18 years of age		PID, SID, ITP, MMN	PID, SID, ITP, CIDP	ITP patients at high risk of bleeding or prior to surgery to correct platelet count
Concentration	5 or 10% upon reconstitution	10%		10%	10%	10%
IgA content	≤ 2.2 μg/mL in 5% solution	Average 0.046 g/L		≤ 0.14 mg per mL	No more than 25 mcg/mL	< 0.4 mg/mL
Storage temparature	Not to exceed +25 °C (77F)	May be stored for 36 months at +2 to +8 °C, AND product may be stored at temperatures not to exceed +25 °C (77°F) for up to 6 months anytime during the 36 month shelf life		Store in refrigerator (+ 2 to + 8 °C) for up to 36 months. Within the first 24 months of the date of manufacture, product may be stored for a single period of up to 12 months at room temperature (below + 25 °C). After this period, unused product must be discarded.	Can be stored either in the refrigerator or at room temperature + 2 to + 25 °C	Store at + 2 to + 8 °C for 23 months from the date of manufacture. Within this shelf-life, product may be stored up to 3 months at ≤ 25 °C. After the storage at ≤ 25 °C the product must be used or discarded
Initial recommended infusion rate	5 or 10% solution: 0.5 mL/kg/h	0.01 to 0 02 mL/kg per min (1 to 2 mg/kg per min) for the first 30 min		0.5 mL/kg BW/hr for 30 min For MMN: 0.5 mL/kg BW/hr	0.5 mg/kg/min (0.3 mL/kg/hr)	1 mg/kg/min (0.01 mL/kg/min) for the first 30 min
Availabe sizes distributed by CBS	5 g/vial	2.5 g/25 mL, 5 g/50 mL, 10 g/100 mL, 20 g, 200 mL	20 g/200 mL	2.5 g/25 mL, 5 g/50 mL, 10 g/100 mL, 20 g/200 mL, 30 g/300 mL	2.5 g/25 mL, 5 g/50 mL, 10 g/100 mL, 20 g/200 mL, 40 g/400 mL	5 g/50 mL, 10 g/100 mL, 20 g/200 mL
Manufacturer website	http://www.baxter.ca	http://www.grifols.com		http://www.baxter.ca	http://www.cslbehring.ca	www.octapharma.ca

Adapted from Canadian Blood Services 2016. Complete tables available at: https://professionaleducation.blood.ca/en/transfusion/clinical-guide/immune-globulin-products

**These products may not be available in all cities/provinces across Canada, and other Ig products not listed here may be available**

*PID* primary immunodeficiency, *SID* secondary immunodeficiency, *ITP* idiopathic thrombocytopenic purpura, *CIDP* chronic inflammatory demyelinating polyneuropathy, *CLL* B-cell chronic lymphocytic leukemia, *MMN* multifocal motor neuropathy, *BW* body weight

The recommended starting dose of Ig replacement therapy is 400–600 mg/kg/4 weeks for the IV formulation and 100–150 mg/kg/week for the subcutaneous formulation [10]. The most common adverse events associated with this therapy are headache, flushing, chills, myalgia, wheezing, tachycardia, lower back pain, nausea, and hypotension. In patients experiencing multiple adverse reactions to one product, consideration may be given to switching to another product or route of administration [10]. Evidence suggests that trough levels should be assessed regularly and that dose may need to be adjusted depending upon the frequency of infection. Lower trough levels have been associated with the progression of chronic lung disease in otherwise asymptomatic patients [34, 35], suggesting that physicians must be diligent in maintaining good levels of serum IgG, and should increase the amount given if there are signs of changing lung function or if the patient continues to experience recurrent infections.

For patients with recurrent infections, prophylactic antibiotic therapy (particularly with agents that provide coverage of *S. pneumoniae* and *H*. *influenzae)* may also be needed in addition to Ig replacement therapy. Depending on the etiology of the specific B-cell disorder, prophylactic antifungal therapy may also be required. Since B-cell immunodeficiencies are often associated with hearing loss and pulmonary complications, regular hearing assessments and monitoring of pulmonary status and function is recommended. As with primary T-cell defects, vigilance for malignancies and autoimmune disorders is also important in patients with B-cell disorders.

At present, there are no definitive management strategies that can be routinely recommended for patients with B-cell disorders. However, gene therapy is currently being investigated for some antibody deficiencies and may represent a future treatment option for these patients [31, 32].

### Innate disorders

The management of innate disorders depends on the type of defect. For phagocyte disorders, therapy is primarily supportive and includes both antibiotic and antifungal prophylaxis. Cytokine replacement (e.g., interferon-γ) and BMT have also been shown to be effective in patients with CGD. Gene therapy may also be a potential definitive treatment option in the future [9, 31, 32].

There is no specific definitive therapy for complement deficiencies. Treatment of these disorders focuses on antibiotic prophylaxis for the prevention of recurrent infections. Since some patients with complement disorders are at increased risk of meningococcal infections with *Neisseria meningitidis,* multivalent meningococcal vaccinations should also be considered (see the *Canadian Immunization Guide for the Vaccination of Immunocompromised Patients with PID* at: https://www.canada.ca/en/public-health/services/publications/healthy-living/canadian-immunization-guide-part-3-vaccination-specific-populations/page-8-immunization-immunocompromised-persons**)** [36]. Pneumococcal and *H. influenzae* vaccines may also be needed in patients with frequent infections caused by encapsulated organisms.

Finally, new biologic therapies, including check point inhibitors, are predicted to influence some of the innate pathways. The use of these biologic therapies in innate deficiencies is under investigation, and these biologics represent potential future therapies for these diseases.

## Prognosis

The prognosis of patients with PIDs varies depending on the etiology of the disorder. However, patient outcomes and long-term survival have improved significantly since the 1970s given our improved management of infections and early access to antibiotics, advances in BMT and HSCT techniques, and enhanced intensive care services. Furthermore, routine vaccinations provide herd immunity to those at risk, decreasing the circulation of infectious disease. Further progress in the diagnosis and management of PIDs is expected as research on the genes responsible for immunodeficiencies and the use of definitive treatments such as gene therapy continues.

## Conclusions

PID refers to a heterogeneous group of disorders that result from defects in immune system development and/or function. PIDs present in both children and adults, and although signs and symptoms are highly variable, most disorders involve increased susceptibility to infection, with many leading to significant disease-associated morbidity and mortality. Other important signs of PID include excessive inflammatory responses and autoimmunity. Given the complexity of these disorders, referral to an immunologist is required for appropriate diagnosis and management. Severe disorders such as SCID require definitive therapy for immune reconstitution (e.g., BMT, HSCT, gene therapy) as soon as possible, which has led to the application of newborn screening to this population. B-cell or antibody-deficiency disorders are the most common types of PIDs. The mainstay of treatment for patients with these disorders is Ig replacement therapy, and there are now several Ig products approved in Canada for patients with PID.

Physicians must be diligent in maintaining good levels of serum IgG since lower trough levels have been associated with the progression of chronic lung disease in otherwise asymptomatic patients. Patients with innate immunodeficiency disorders often present with unusual or difficult to eradicate infections. Treatment varies depending on the type of defect (e.g., phagocyte disorder or complement deficiency), but may involve antifungal and antibiotic prophylaxis, cytokine replacement, vaccinations and BMT.

## Key take-home messages

- With the exception of IgA deficiency (prevalence = 1 in 300–500), PIDs are more frequent than previously believed, with an estimated prevalence of 1 in 1200 live births.
- Clinical presentation is highly variable, but most disorders involve increased susceptibility to infection. Excessive inflammatory responses and autoimmunity are also common manifestations of immune deficiency.
- PIDs should be suspected at any age in patients with: recurrent sinus or ear infections or pneumonias within a 1 year period; failure to thrive; poor response to prolonged use of antibiotics; persistent thrush or skin abscesses; or a family history of PID.
- Consultation with a clinical immunologist is required to confirm the diagnosis of PID and to establish an appropriate treatment plan.
- SCID is fatal unless the underlying defect is corrected and, therefore, definitive therapy with HSCT should be initiated as quickly as possible. The importance of prompt diagnosis has led to newborn screening for SCID using the TREC assay, which is available in some centres.
- Ig replacement therapy is the mainstay of therapy for antibody-deficiency disorders, and is also an important supportive treatment for many patients with other forms of PID including SCID/CID. Several Ig replacement products are now available in Canada.
- Antibiotic and antifungal prophylaxis are also recommended for many PIDs to prevent the frequency and severity of infections.

## Abbreviations

PID: primary immunodeficiency disorder
Ig: immunoglobulin
SCID: severe combined immunodeficiency
CIDs: combined (B- and T-cell) immunodeficiency disorders
NK: natural killer
HCST: hematopoietic stem cell transplantation
LOCID: late-onset combined immunodeficiency
IV: intravenous
ADA: adenosine deaminase
HLA: human leukocyte antigen
TMP-SMX: trimethoprim-sulfamethoxazole
STAT3: signal transducer and activator of transcription 3
DHR: dihydrorhodamine 1,2,3 response
TREC: T-cell receptor excision circle
CGD: chronic granulomatous disease
XLA: X-linked agammaglobulinemia
Btk: Bruton’s tyrosine kinase
CVID: common variable immunodeficiency
CBC: complete blood count
BMT: bone marrow transplantation
HLH: hemophagocytic lymphohistiocytosis
ALPS: autoimmune lymphoproliferative syndrome
IPEX: immunodysregulation polyendocrinopathy enteropathy X-linked
APECED: autoimmune polyendocrinopathy candidiasis and ectodermal dystrophy
